# OA02 A five-decade journey through x-linked hypophosphataemia: a case report

**DOI:** 10.1093/rap/rkag113.002

**Published:** 2026-11-03

**Authors:** Arjun Narayana, Kirishananth Rajaseelan, Anupama Nandagudi

**Affiliations:** Mid and South Essex NHS Foundation Trust, Basildon, United Kingdom; Mid and South Essex NHS Foundation Trust, Basildon, United Kingdom; Mid and South Essex NHS Foundation Trust, Basildon, United Kingdom

## Abstract

**Introduction:**

Hypophosphataemic rickets (HR) is a rare inherited disorder, most commonly X-linked hypophosphataemia (XLH), characterised by renal phosphate wasting and impaired bone mineralisation. It typically presents in early childhood with skeletal deformities such as bow legs, short stature, and growth-plate abnormalities. Around 800 individuals in the UK are affected. Management remains challenging due to the absence of curative therapy and multiple long-term sequelae including deformity, pain, and fracture risk. This case report describes a 48-year-old woman with HR followed over five decades, illustrating the evolving complexity of surveillance, medication adjustment, and electrolyte management throughout her life.

**Case description:**

Our patient is a 48-year-old woman with HR, presenting in childhood with short stature, genu valgum, and scoliosis. A skeletal survey revealed delayed bone maturation, with skeletal age approximately three years behind chronological age. Diagnostic uncertainty in the 1980s led to referral to a regional specialist in growth disorders. Investigations confirmed hypophosphataemia (0.6 mmol/L) and a high phosphate excretion index (0.36), consistent with renal phosphate wasting. Treatment commenced with phosphate supplementation 250 mg five times daily and calcitriol 0.25 micrograms BD.

Over the subsequent years, electrolyte levels normalised, and growth improved to age-appropriate height, though genu valgum persisted, limiting mobility. Four years later, she developed malaise and hypercalcaemia, prompting close biochemical monitoring and temporary cessation of calcitriol, later reintroduced at two-thirds of the original dose.

In adulthood, she developed genu varum and radiological evidence of a healed femoral shaft fracture with a persistent Looser’s Zone. In her thirties, discontinuation of supplements led to worsening mobility, bilateral hip and groin pain, and multiple falls. Imaging demonstrated widespread skeletal sclerosis without evidence of osteoporosis on DEXA scanning. She sustained a new metatarsal fracture, and her son was subsequently diagnosed with HR, confirming familial inheritance.

During her forties, treatment with alfacalcidol 1 microgram OD was used to stabilise calcium and phosphate levels. A short course of denosumab was trialled to address bone pain and fragility, with biochemical improvement but limited functional recovery.

**Discussion:**

This case report highlights the lifelong complexity of managing HR and the essential role of a multidisciplinary team in optimising outcomes. Management must address biochemical, skeletal, and genetic aspects while anticipating complications from long-term therapy.

**Diagnostic Advances and Genetic Counselling** Since this patient’s childhood diagnosis, identification of PHEX mutations has enabled genetic confirmation and family screening. XLH follows an X-linked dominant inheritance pattern—mothers can transmit the condition to both sons and daughters, while affected fathers pass it only to daughters. Disease severity appears independent of sex. Advances in prenatal and preimplantation genetic testing now allow early diagnosis and informed reproductive counselling.

**Osteoporosis and Bone Health** Reduced bone mineralisation in XLH complicates DEXA interpretation, as low density may reflect osteomalacia rather than osteoporosis. Conventional anti-resorptive agents such as bisphosphonates do not correct underlying mineralisation defects and may exacerbate Looser’s zones. Their use should be reserved for confirmed co-existing osteoporosis following multidisciplinary evaluation.

**New Targeted Treatments: burosumab** The discovery of PHEX gene mutations and their link to increased Fibroblast Growth Factor-23 (FGF-23) activity has transformed understanding of XLH. Excess FGF-23 suppresses renal phosphate reabsorption and impairs vitamin D activation, perpetuating hypophosphataemia and defective mineralisation. Burosumab, a monoclonal antibody against FGF-23, restores phosphate and vitamin D homeostasis and has demonstrated safety and efficacy in improving skeletal outcomes. Its introduction represents a major advance in targeted therapy for HR, though long-term data on durability and lifetime effects remain limited.

**Multidisciplinary Approach and Long-Term Care** Overall, this case underscores the need for lifelong, coordinated care integrating endocrinology, orthopaedics, genetics, and metabolic expertise.

**Key learning points:**

**Diagnosis and Genetic Counselling** Early recognition of hallmark features—short stature, bow legs, and impaired mobility—is crucial for timely diagnosis and initiation of phosphate supplementation and, where appropriate, Burosumab therapy. Advances in genetic testing now enable prenatal and preimplantation diagnosis, allowing early intervention before skeletal deformities develop. Genetic counselling remains essential for families affected by XLH to guide reproductive decisions and anticipate disease severity.

**Management of Complications** Fractures, skeletal deformities, and chronic pain significantly impair mobility and quality of life. Regular monitoring of bone biochemistry and phosphate supplementation are key to maintaining bone health. Secondary hyperparathyroidism and dental abnormalities should be actively screened and managed. Multidisciplinary involvement—including endocrinology, orthopaedics, and physiotherapy—is vital for optimising outcomes and preserving function.

**Co-existing Osteoporosis** DEXA interpretation in HR is challenging due to reduced bone mineralisation and structural variation. Osteoporosis treatments such as bisphosphonates are generally contraindicated, as they may exacerbate hypophosphataemia and worsen osteomalacia. Therapy should only be considered following multidisciplinary confirmation of true co-existing osteoporosis.

**Burosumab therapy** Burosumab, a monoclonal antibody targeting FGF-23, represents a major advancement in HR management. By correcting phosphate wasting and improving vitamin D metabolism, it offers potential for long-term skeletal and functional improvement. Ongoing research is needed to define optimal treatment duration, lifetime safety, and its role in preventing late-stage complications.

**Specialist MDT Discussions** MDT reviews allow individualised treatment planning, balancing biochemical control, skeletal health, and comorbidity management. Such collaboration also supports the development of shared protocols and contributes to the growing evidence base for rare bone disease care.

